# Single-cell RNA sequencing highlights the immunosuppression of IDO1^+^ macrophages in the malignant transformation of oral leukoplakia

**DOI:** 10.7150/thno.99112

**Published:** 2024-08-12

**Authors:** Yu Zhang, Jie Zhang, Simin Zhao, Yan Xu, Yingying Huang, Shaopeng Liu, Peng Su, Caijiao Wang, Yahui Li, Hao Li, Pishan Yang, Chengzhe Yang

**Affiliations:** 1 School and Hospital of Stomatology, Cheeloo College of Medicine, Shandong University & Shandong Key Laboratory of Oral Tissue Regeneration & Shandong Engineering Research Center of Dental Materials and Oral Tissue Regeneration & Shandong Provincial Clinical Research Center for Oral Diseases, Jinan, Shandong, China.; 2 Department of Oral and Maxillofacial Surgery, Qilu Hospital of Shandong University, Jinan, Shandong, China.; 3 Advanced Medical Research Institute, Shandong University, Jinan, Shandong, China.; 4 Jinan Stomatological Hospital, Jinan, Shandong, China.; 5 Department of Stomatology, Shandong Provincial Hospital Affiliated to Shandong First Medical University & Department of Stomatology, Shandong Provincial Hospital, Shandong University, Jinan, Shandong, China.; 6 Department of Pathology, Qilu Hospital of Shandong University, Jinan, Shandong, China.; 7 Department of Pathology, School and Hospital of Stomatology, Cheeloo College of Medicine, Shandong University & Shandong Key Laboratory of Oral Tissue Regeneration & Shandong Engineering Research Center of Dental Materials and Oral Tissue Regeneration & Shandong Provincial Clinical Research Center for Oral Diseases, Jinan, Shandong, China.; 8 Department of Periodontology, School and Hospital of Stomatology, Cheeloo College of Medicine, Shandong University & Shandong Key Laboratory of Oral Tissue Regeneration & Shandong Engineering Research Center of Dental Materials and Oral Tissue Regeneration & Shandong Provincial Clinical Research Center for Oral Diseases, Jinan, Shandong, China.

**Keywords:** Oral squamous cell carcinoma, Oral leukoplakia, immunosuppressive microenvironment, macrophage, indoleamine 2, 3-dioxygenase 1.

## Abstract

**Rationale**: Immunosuppressive tumor microenvironment (iTME) plays an important role in carcinogenesis, and some macrophage subsets are associated with iTME generation. However, the sub-population characterization of macrophages in oral carcinogenesis remains largely unclear. Here, we investigated the immunosuppressive status with focus on function of a macrophage subset that expressed indoleamine 2,3 dioxygenase 1 (Macro-IDO1) in oral carcinogenesis.

**Methods**: We built a single cell transcriptome atlas from 3 patients simultaneously containing oral squamous cell carcinoma (OSCC), precancerous oral leukoplakia (preca-OLK) and paracancerous tissue (PCA). Through single-cell RNA sequencing and further validation using multicolor immunofluorescence staining and the *in vitro*/*in vivo* experiments, the immunosuppressive cell profiles were built and the role of a macrophage subset that expressed indoleamine 2,3 dioxygenase 1 (Macro-IDO1) in the malignant transformation of oral leukoplakia was evaluated.

**Results**: The iTME formed at preca-OLK stage, as evidenced by increased exhausted T cells, Tregs and some special subsets of macrophages and fibroblasts. Macro-IDO1 was predominantly enriched in preca-OLK and OSCC, distributed near exhausted T cells and possessed tumor associated macrophage transformation potentials. Functional analysis revealed the established immunosuppressive role of Macro-IDO1 in preca-OLK and OSCC: enriching the immunosuppression related genes; having an established level of immune checkpoint score; exerting strong immunosuppressive interaction with T cells; positively correlating with the CD8-exhausted. The immunosuppression related gene expression of macrophages also increased in preca-OLK/OSCC compared to PCA. The use of the IDO1 inhibitor reduced 4NQO induced oral carcinogenesis in mice. Mechanistically, IFN-γ-JAK-STAT pathway was associated with IDO1 upregulation in OLK and OSCC.

**Conclusions**: These results highlight that Macro-IDO1-enriched in preca-OLK possesses a strong immunosuppressive role and contributes to oral carcinogenesis, providing a potential target for preventing precancerous legions from transformation into OSCC.

## Introduction

Oral squamous cell carcinoma (OSCC) is a common malignancy tumor derived from oral mucosal epithelium. More than 380 000 new cases were reported in 2022 worldwide accompanied by gradually increasing tendency and poor prognosis 1. The main treatment modality for OSCC is a comprehensive sequence of mainly surgical treatments; however, 1.9% still die from oral cancer each year 1. One of the main reasons for the poor prognosis of oral cancer is being diagnosed at an advanced stage, although such diseases are typically exposed in the oral cavity and easily detected. Thus, exploring the early events in OSCC initiation is important for its early prevention and treatment.

Oral leukoplakia (OLK) is a frequent oral mucosal disease with a 4.11% of prevalence and has been considered as precancerous lesion 2. OLK can transform into OSCC under affection of various complex factors with ~20% of transformation rate 3-5. Convincing evidence shows that immunosuppression is not only immunological features of OSCC but also is an important event in the transformation of OLK to OSCC. Tumor associated macrophages (TAMs), one of the important immunosuppressive cells, are associated with the immunosuppressive status of TME, including exhausted T cell generation, cytotoxic T cell dysfunction, anti-PD-1/PD-L1 resistance 6, 7, among the others. Furthermore, macrophage polarization also exerts obvious affection on carcinogenesis. In OLK, CD163^+^macrophages (M2) infiltration correlates with IL-10 expression 8. However, TAMs display remarkable plasticity within the TME and may transform from one phenotype to another and they always present a mixture of M1-like and M2-like phenotypes 9. Therefore, widely used M1/M2 dichotomy *in vitro* may not necessarily reflect the complexity of TAMs *in vivo*. With the single-cell RNA sequencing technologies development, more macrophage subpopulations have been identified, including the SPP1^+^TAMs associated with angiogenesis in colon cancer 10, TREM2^+^TAMs related to immunosuppressive TME 11, and PD-L1^+^macrophage mediating immune evasion in melanoma 12. However, predominant subsets of TAMs responsible for transformation of OLK to OSCC have been few studied until recently 4, 13.

In this study, using newly generated scRNA-seq data of tumor, precancerous OLK (preca-OLK) and paracancerous tissue (PCA) derived from 3 cases of OSCC patients transformed from OLK, the overall and special cell atlas in the three tissues were obtained and the proportion of various cell types compared. Then, we put focus on IDO1^+^macrophages subset and systematically investigated its role in promoting the conversion of OLK to OSCC through immunosuppression.

## Materials and Methods

### Collection of clinical human samples

With informed written consent, adjacent normal mucosa, oral leukoplakia and tumor tissues were collected from OSCC patients with a history of oral leukoplakia. Fresh tissues were preserved in MACS® Tissue Storage Solution (Miltenyi Biotec, Cat# 130-100-008) for transport. The experiment was approved by the Ethics Committee of Qilu Hospital, Shandong University, China.

### Preparation of single-cell suspensions of tissue samples

Under aseptic conditions, the tissue was washed twice with precooled RPMI 1640+0.04% BSA medium. The tissues were finely dissected and then placed into freshly prepared enzyme digestion solution (0.02% collagenase, Type II, Gibco, 17101015), followed by enzymatic digestion for 60 minutes at 37°C. Subsequently, the digested cell suspension was filtered through a BD 40 μm cell sieve and subsequently centrifuged before being resuspended in an equal volume of erythrocyte lysate (MACS, Art. No. 130-094-183). After washing with medium, dead cells were removed using the MACS Dead Cell Removal Kit (130-090-101) according to the manufacturer's instructions. The processed samples were subjected to cell viability testing, cell counting, and single-cell isolation.

### Single-cell RNA sequencing

Single-cell RNA sequencing was performed using the BD RhapsodyTM platform. Following the manufacturer's recommendations, the single-cell suspension was adjusted to the appropriate volume for loading and capture using the BD Rhapsody™ Enhanced Cartridge Reagent Kit (Cat. No. 664887) and BD Rhapsody™ Cartridge Kit (Cat. No. 633733). Reverse transcription was performed using a BD Rhapsody™ cDNA kit (Cat. No. 633773). A BD Rhapsody™ WTA Amplification Kit (Cat. No. 633801) was used for DNA library construction. High-throughput sequencing was performed in PE-150 mode.

### Sequencing data quality control and gene quantification

The FASTQ files were processed and aligned to the human reference genome using the BD Rhapsody WTA Analysis Pipeline (version 2.0) with unique molecular identifier (UMI) counts summarized for each barcode. The UMI count matrix was then analyzed using Seurat 14 (version 4.0.0) R package. To remove low-quality cells and likely multiplet captures, a set of criteria were conducted: Cells were filtered by (1) gene numbers (gene numbers < 200), (2) UMI (UMI <1000), (3) log10GenesPerUMI (log10GenesPerUMI < 0.7), (4) percentage of mitochondrial RNA UMIs (proportion of UMIs mapped to mitochondrial genes > 10%) and (5) percentage of hemoglobin RNA UMIs (proportion of UMIs mapped to hemoglobin genes > 5%). Subsequently, we applied the DoubletFinder package 15 (version 2.0.3) to assess potential doublets and multiplets. To obtain the normalized gene expression data, library size normalization was implemented by the NormalizeData function. Specifically, the global-scaling normalization method “Log-Normalize” normalized the gene expression measurements for each cell by the total expression, multiplied by a scaling factor (10,000 by default), and log-transformed the results.

### Downscaling and cluster analysis

The top 2000 highly variable genes (HVGs) were calculated using the Seurat function FindVariableGenes (mean.function = FastExpMean, dispersion.function = FastLogVMR). To eliminate the batch effect, we corrected the batch effect of single-cell expression profiling data by using the mutual nearest neighbors (MNN) method in the batchelor (version 1.6.3) package 16. Principal component analysis (PCA) was performed to reduce the dimensionality with the RunPCA function. Graph-based clustering was performed to cluster cells according to their gene expression profile with the FindClusters function. Cells were visualized using a 2-dimensional Uniform Manifold Approximation and Projection (UMAP) algorithm with the RunUMAP function.

### Marker gene identification

The FindAllMarkers function (test.use = presto) was used to identify marker genes of each cluster. The identified marker genes were visualized by the VlnPlot and FeaturePlot functions.

### Differential expression analysis

We used the “FindAllMarkers” function in Seurat to identify genes that were differentially expressed between clusters with the following parameters: only.pos = T, logfc.threshold = 0, min.pct = 0.25, and pseudocount.use = 1. The nonparametric Wilcoxon rank-sum test was used to obtain p values for comparisons, and the adjusted p values, based on Bonferroni correction, were calculated for all genes in the dataset. We used a heatmap to visualize DEGs based on gene expression after log transformation and scaling. GO and KEGG enrichment analyses of DEGs were performed by hypergeometric distribution tests.

### Definition of cell scores and signatures

We used the function addmodule score in Seurat to calculate the average single-cell expression levels of each programme. All analyzed features are binned Based on average expression, and the control features are randomly selected from each bin. The gene sets associated with the abovementioned genes are shown in Table S1.

### Gene Set Variation Analysis (GSVA)

To perform the gene set variation analysis, the GSEABase package (version 1.44.0) was used to load the gene set files, which were downloaded and processed from the KEGG database (https://www.kegg.jp/). To assign pathway activity estimates to individual cells, we applied GSVA 17 using standard settings, as implemented in the GSVA package (version 1.30.0). The differences in pathway activity per cell were calculated with the LIMMA package (version 3.38.3).

### Monocle2 Pseudotime Analysis

The developmental pseudotime was determined with the Monocle2 18 package (version 2.9.0). The raw count was first converted from the Seurat object into the CellDataSet object with the importCDS function in Monocle. The differentialGeneTest function of the Monocle2 package was used to select ordering genes (qval < 0.01) that were likely to be informative in the ordering of cells along the pseudotime trajectory. Dimensional reduction clustering analysis was performed with the reduceDimension function, followed by trajectory inference with the orderCells function using default parameters. Gene expression was plotted with the plot_genes_in_pseudotime function.

### Cell Communication Analysis

The CellPhoneDB 19 (version 4.1.0) was used to identify biologically relevant ligand‒receptor interactions from single-cell transcriptomics (scRNAseq) data. A ligand or a receptor was defined as “expressed” in a particular cell type if 10% of the cells of that type had nonzero read counts for the ligand/receptor encoding gene. Statistical significance was then assessed by randomly shuffling the cluster labels of all cells and repeating the above steps, which generated a null distribution for each LR pair in each pairwise comparison between two cell types. After running 1,000 permutations, P values were calculated with the normal distribution curve generated from the permuted LR pair interaction scores. To define networks of cell‒cell communication, any two cell types where the ligand was expressed in the former cell type and the receptor in the latter was linked. R packages Igraph and Circlize were used to display the cell‒cell communication networks.

### CellChat Analysis

Cell communication analysis was performed using CellChat 20 (version 1.1.3) R package. First, we imported the normalized expression matrix to create the cell chat object with the createCellChat function. Second, the data were preprocessed with the identifyOverExpressedGenes, identifyOver-ExpressedInteractions and projectData functions using the default parameters. The compute-CommunProb, filterCommunication (min.cells = 10) and computeCommunProbPathway functions were then used to determine any potential ligand‒receptor interactions. Finally, the cell communication network was aggregated using the aggregateNet function.

### SCENIC Analysis

SCENIC analysis was performed using the motif database for RcisTarget and GRNboost (SCENIC 21 version 1.2.4, which corresponds to RcisTarget version 1.10.0 and AUCell version 1.12.0) with the default parameters. In detail, transcription factor (TF) binding motifs overrepresented on a gene list were identified with the RcisTarget package. The activity of each group of regulons in each cell was scored by the AUCell package (version 1.12.0).

To evaluate the cell type specificity of each predicted regulon, the regulon specificity score (RSS), which was based on the Jensen‒Shannon divergence (JSD), a measure of the similarity between two probability distributions, was calculated. Specifically, the JSD (Jensen-Shannon divergence) between each vector of binary regulon activity overlaps with the assignment of cells to a specific cell type 22 was calculated. The connection specificity index (CSI) for all regulons was calculated with the scFunctions (https://github.com/FloWuenne/scFunctions/) package.

### Gene Set Enrichment Analysis (GSEA)

Gene Set Enrichment Analysis (GSEA 23) was used to complete Gene Ontology (GO) and KEGG term enrichment analysis with the Molecular Signatures Database (MSigDB) C5 GO gene sets and C2 KEGG gene sets (version 7.2) separately.

### Analyses of The Cancer Genome Atlas (TCGA) database

Transcriptome data from the TCGA_HNSCC dataset were retrieved from UCSC Xena (https://xena.ucsc.edu/). Analyses were performed on the GEPIA2 website (gepia2.-cancer-pku.cn/) 24.

### GEO database analysis

All microarray data were downloaded from the GEO database (https://www.ncbi.nlm.nih.gov/geo/). The raw data were downloaded as MINiML files. Box plots were drawn by boxplot; the R software ggord package was used to draw PCA plots. The R software ggstatsplot package was used to draw two-gene correlations. Spearman's correlation analysis was used to describe the correlation between quantitative variables without a normal distribution. P values less than 0.05 were considered to indicate statistical significance (*P < 0.05).

### Multiple immunofluorescences staining for clinical samples

Paraffin tissue sections of 25 patients with oral leukoplakia (OLK) and 15 patients with leukoplakia malignant to oral squamous cell carcinoma who underwent surgery at Qilu Hospital of Shandong University between January 2020 and December 2022 were selected. All pathological states were confirmed histopathologically by H&E staining. Multiple immunofluorescence staining (mIFC) of tissue was performed using Opal Chemistry (PerkinElmer, Waltham, MA, USA). Briefly, the sections were labeled with primary antibodies anti-IDO1 (Proteintech, 13268-1-AP), anti-CD68 (Abcam, ab955), anti-Pan-CK (ZSGB-BIO, ZM-0069), anti-CD3 (ZSGB-BIO, ZM-0417), anti-CD8 (ZSGB-BIO, ZA-0508), and anti-PD-1 (ZSGB-BIO, ZM-0381), followed by HRP-conjugated secondary antibody. Subsequently, the fluorophore-conjugated tyramide amplification system (PerkinElmer) was used for signal amplification, and DAPI was used to counterstain the nuclei. Visualization and quantitation of the different fluorophores were achieved with Tissue FAXS Spectra Systems and Strata Quest analysis software (Tissue Gnostics).

### Cell lines and IFN-γ treatment

The human monocytic cell line THP-1 was cultured in RPMI 1640 (Gibco) medium supplemented with 10% FBS. The THP-1 cell line was treated for 24 h with different concentrations of IFN-γ (Yeasen, 91207ES60) (0-60 ng/mL).

### Quantitative real-time polymerase chain reaction (qRT‒PCR) analysis

A Fastagen Biotech™ RNAfast200 Extreme Total RNA Extraction Kit (Fastagen) was used to extract RNA, and Hifair® III 1st Strand cDNA Synthesis SuperMix for qPCR (gDNA digester plus) (YEASEN) was used to reverse transcribe 1000 ng of total RNA into complementary DNA (cDNA) in a total volume of 20 µl. Subsequently, RT‒qPCR was performed on an Analytik Jena-gTower 3G instrument using Hieff® qPCR SYBR Green Master Mix (No Rox) (YEASEN). The sequences of the primers used were as follows (5'-3'): H-IDO1-80F, AGTCAAATCCCTCAGTC-CGTG and H-IDO1-80R, TTTCACACAGGCG-TCATAAGC; H-GAPDH-138F, GCACCGTCAAG-GCTGAGAAC and H-GAPDH-138R, TGGTGAAG-ACGCCAGTGGA (Azenta, USA). The PCR amplification procedure included incubation at 95°C for 30 s, 45 cycles of denaturation at 95°C for 10 s and extension at 60°C for 30 s. Each sample was run in triplicate, and the values were calculated according to the 2-ΔΔCt equation.

### Mice

All animal studies were approved by the Laboratory Animal Ethics Committee of Shandong University (Permit No. 23047). Thirty-six six-week-old male C57BL/6 mice purchased from GemPharmatech were divided into the untreated group, IDO1i group, anti-PD-1 group, and IDO1i+anti-PD-1 group, with 9 mice in each group. All mice were given sterile water containing 100 μg/mL 4NQO (Sigma Aldrich, N8141) for 16 consecutive weeks. From week 17 to week 22, all mice returned to normal drinking water. Epacadostat (Selleck, INCB024360), an IDO1 inhibitor, was dissolved in 1% CMC-Na and administered by oral gavage at a dose of 4 mg per animal per day for 5 consecutive days per week after a 2-day interval. Anti-PD-1(Bio X-Cell, clone RMP1-14) was administered intraperitoneally at a dose of 200 μ g to each animal twice a week. The untreated group was administered 1% CMC-Na by oral gavage 2 days after 5 consecutive days of weekly dosing. All mice were dosed from 12 to 15 weeks after induction, and the experiments continued until the end of week 22 and were conducted in a specific pathogen-free environment (23°C, 60% humidity). Mice were euthanized at week 22, and tongue lesions were harvested for histopathological analysis.

### Statistical analysis

All statistical analyses were performed using GraphPad Prism 8 software and R language (v3.6.2). Independent- sample Student's t-test was used for comparison between two experimental groups. The error bars in the figures represent the mean ± sem. P value of less than 0.05 was considered statistically significant, defined as *P < 0.05, **P < 0.01, ***P < 0.001.

## Results

### Global transcriptomic landscape and the proportion change of immune cells in oral precancer and cancer tissues

**Figure 1:**
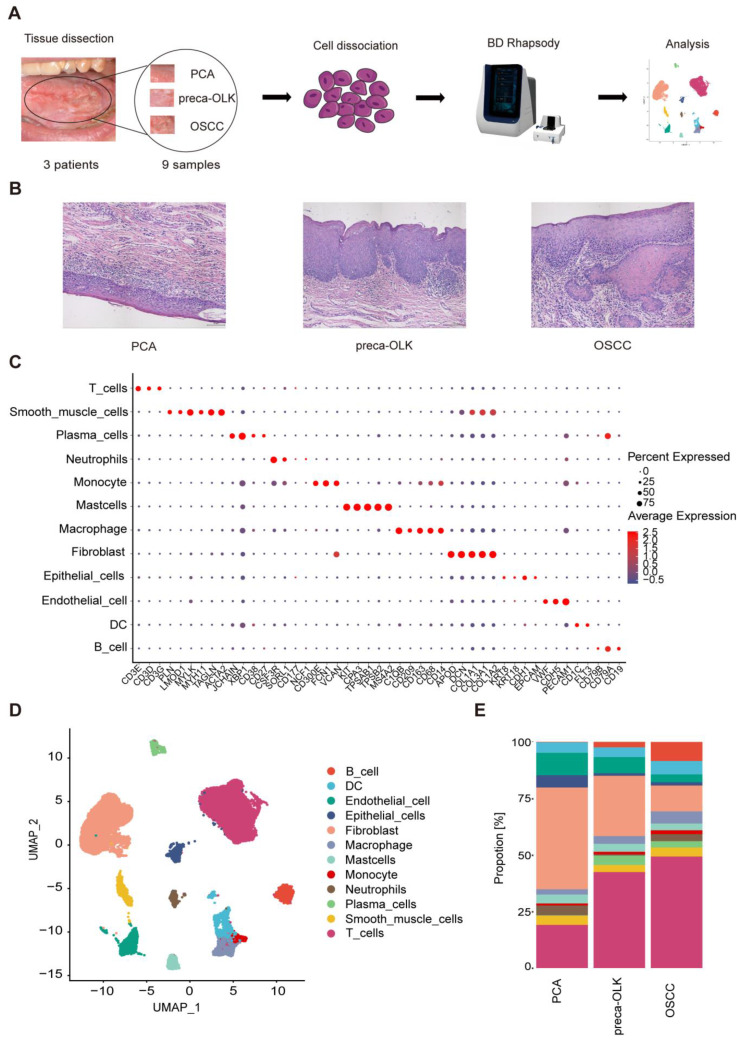
** Single-cell transcriptomic landscape of paired human PCA, preca-OLK, and OSCC tissues (A)** Overview of the workflow and the experimental design for scRNA-Seq. **(B)** Representative H&E staining of tissue samples biopsied for scRNA-seq. Scale bar: 100μm. **(C)** Dot plot showing the highly expressed marker genes in each major cell type. **(D)** UMAP plot showing the clustering results of 12 major cell types for 42291 high-quality single cells from all the samples. **(E)** Stacked histogram showing the percentages of individual cell types/total cells from PCA, preca-OLK, and OSCC tissues.

Three patients with OSCC from OLK transformation and without any the other tumors, who has not received chemotherapy and radiation therapy or other special treatments, were recruited and 9 tissue samples from these patients simultaneously containing OSCC, OLK being adjacent to OSCC (preca-OLK) and matched PCA 3 cm distant from OSCC were obtained. The biopsies of PCA, preca-OLK and OSCC from each individual were processed separately and the scRNA-seq was performed (Figure 1A) after cell number assessment and pathology confirmation (Figure 1B). After data preprocessing, sample integration and principal component analysis (PCA), a total of 42291 high quality cells, of which 14408, 16827 and 11056 were derived from OSCC, preca-OLK and PCA, respectively, were unsupervisedly clustered into 12 major clusters by applying graph-based uniform manifold approximation and projection (UMAP) method and annotated by cluster-specific marker genes as T cells (CD3G, CD3D, CD3E), smooth muscle cells (ACTA2, TAGLN, MYH11, MYLK, LMOD1, PLN), plasma cells (CD27, CD38, XBP1, JCHAIN), neutrophils (NCF1, CD177, SORL1, CSF3R), monocytes(CD300E, FCN1, VCAN), mast cells (MS4A2, TPSB2, TPSAB1, CPA3, KIT), macrophages (CD14, CD68, CD163, CD209, C1QB), fibroblasts (COL1A2, COL3A1, COL1A1, DCN, APOD), epithelial cells (EPCAM, CDH1, KRT18, KRT8), endothelial cells (PECAM1, CDH5, VWF), dendritic cells (DC) (FIT3, CD1C) and B cells (CD19, CD79A,CD79B) (Figure 1C). The total (Figure 1D) and PCA, preca-OLK and OSCC special (Figure S1) cell atlas were created.

The proportion change of various cell types among PCA, preca-OLK and OSCC tissues was compared and result showed that the proportions of non-immune cells such as fibroblasts, epithelial cells and endothelial cells gradually declined while immune cells including macrophages, T cells, B/Plasma cells gradually increased from PCA to preca-OLK and to OSCC (Figure 1E). These findings suggest that immunoreaction plays the key role in OLK progression to OSCC, consistent with previous reports 13, 25, 26.

### The function and pseudo temporal analysis of fibroblast sub-clusters

**Figure 2:**
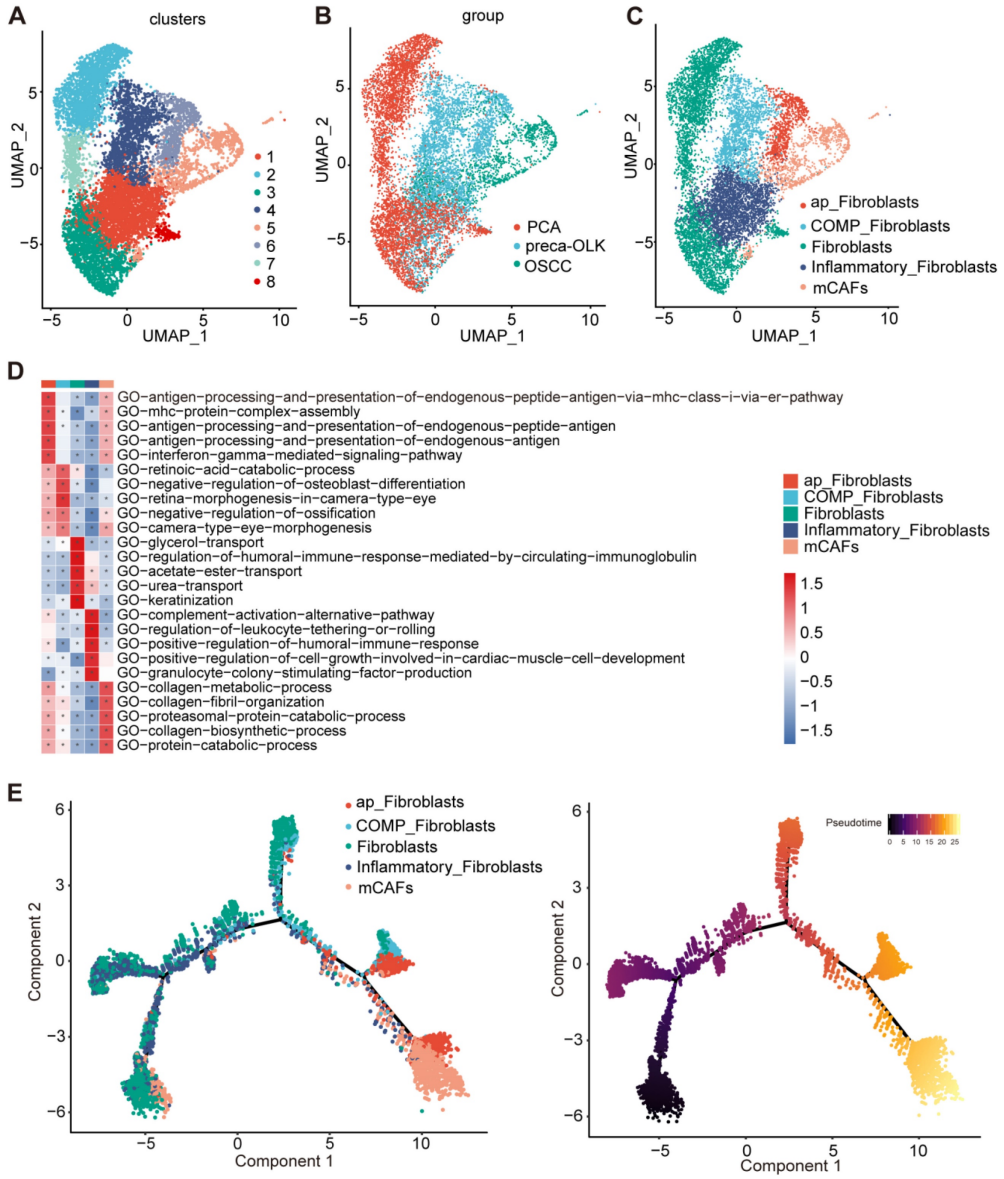
** The function enrichment and differentiation trajectory of fibroblast subpopulations (A)** UMAP plot of the total fibroblast subsets. **(B)** UMAP plot showing the distribution of all fibroblasts in PCA, preca-OLK, OSCC. **(C)** UMAP plots showing the subpopulations of fibroblasts. **(D)** Heatmap showing representative GO pathways enrichment of the gene set expressed in fibroblasts subsets predicted by GSVA. **(E)** UMAP plot showing the differentiation trajectory of fibroblasts and the distribution of each cell subset on the trajectory.

Fibroblasts, the most common cell type among nonimmune cells in TME, play an important role in TME remodeling and tumor development 27. We first performed re-clustering of fibroblasts to obtain 8 sub-sets (Figure 2A). According to distribution and proportions in different tissues (Figure 2B) and gene expression characteristics, these sub-sets were respectively defined (Figure 2C). C2/C3/C7/C8 were mainly found in PCA and were grouped as Fibroblasts. C4/C6 mainly existed in preca-OLK, named as OLK associated fibroblasts. Moreover, C4 overexpressed COMP (Figure S2A), entitled as COMP-Fibroblasts.C6 overexpressed antigen presentation-associated genes such as HLA-DRA, HLA-DRB1, HLA-DPB1 and HLA-DQA1 (Figure S2B), thus, defined by antigen presentation fibroblasts (ap-Fibroblasts). C5 was a subset in OSCC and thus considered to be related to tumor occurrence and development. Further, C5 highly expressed MMP1, MMP11, POSTN, MMP14, ACTA2 and TAGLN, the typical matrix CAFs markers (Figure S2A, Figure S2C) 28, thus named as matrix CAFs (mCAFs). POSTN-encoded protein can promote cancer stem cells stemness maintance and support the adhere and migration of epithelial cells 29. C1 presented in three tissues and highly expressed inflammation-associated genes such as ADH1B, SLPI, CXCL12 and IGF1 (Figure S2D), therefore, designated by Inflammatory Fibroblasts. This reflects heterogeneity of fibroblasts and potential pathogenic role of ap-Fibroblasts, COMP-Fibroblasts in malignancy transformation of OLK.

GSVA GO functional enrichment (Figure 2D) revealed that ap-Fibroblasts enriched in antigen presentation-associated function; Inflammatory Fibroblasts enriched in complement activation and immune response; mCAFs enriched in collagen metabolism and proteasome protein breakdown metabolism process related functions; Moreover, two OLK-associated subsets, COMP-Fibroblasts and ap-Fibroblasts, had similar functions to mCAFs and high enrichment score in mCAFs enrichment pathway. The pseudo temporal analysis of fibroblast sub-clusters performed by monocle2 demonstrated a gradual evolution from initial Fibroblasts and Inflammatory Fibroblasts to intermediate OLK-associated fibroblasts (ap-Fibroblasts and COMP-Fibroblasts) and then to final mCAFs (Figure 2E). These results suggest that some fibroblast subsets in OLK have possessed CAF transformation potentials.

### T cells display gradual dysfunctionality in oral precancer and cancer tissues

**Figure 3:**
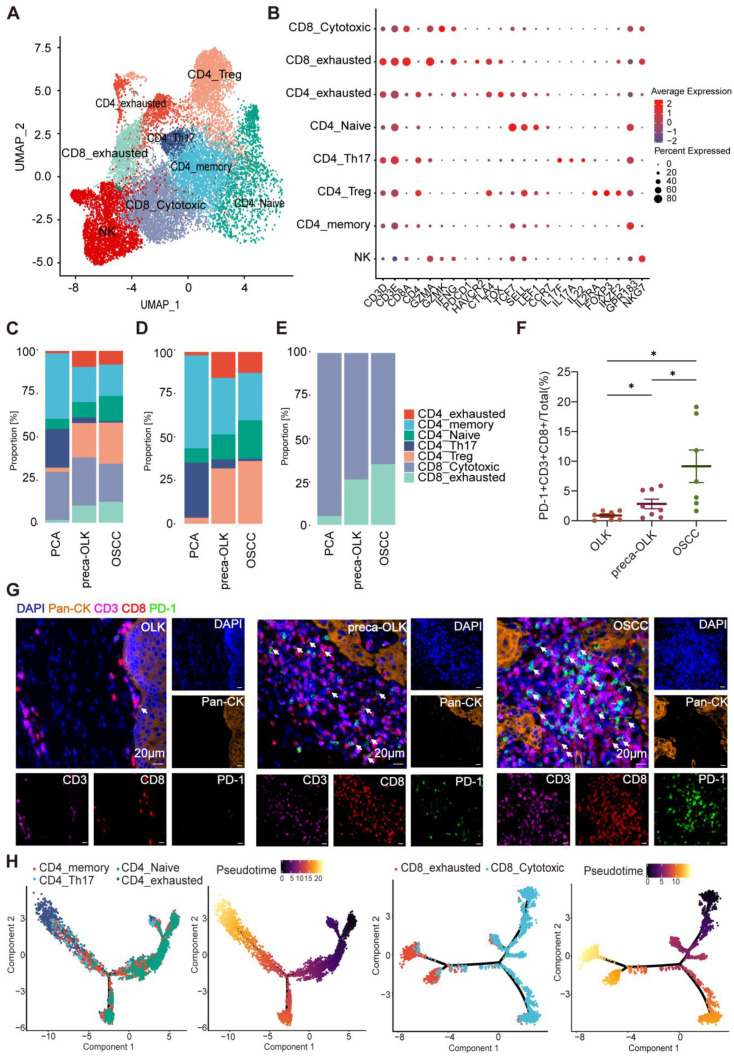
**T cell dysfunction and cell state transitions in carcinogenesis (A)** UMAP plot showing the distribution of T cell subsets. **(B)** Dot plot showing expression of relative markers in indicated T cell clusters. **(C)** Proportion of T cell subtypes was shown in bar plots in different tissues. **(D-E)**. Proportion of CD4^+^T cells **(D)** or CD8^+^T cells **(E)** subtypes were shown in bar plots in different tissues respectively. **(F)** Statistical analysis comparing the proportions of PD-1^+^CD3^+^CD8^+^cells (exhausted CD8^+^T cells) in tissues of OLK (n = 7), preca-OLK (n = 8) and OSCC (n = 7). **(G)** Representative IF staining of human tissues (40x). DAPI (blue), Pan-CK (orange), CD3(purple), CD8(red), PD-1(green). Bar, 20 μm. (H) UMAP plot showing the differentiation trajectory of CD4^+^T cells or CD8^+^T cells and the distribution of each cell subset on the trajectory.

T cells within tumors present heterogeneity and different T cell subsets display varied effects 30. To investigate the cellular state and function of T cells in the TME or in OLK, we performed re-clustering of T cells to obtain UMAP figure of T cells (Figure 3A). According to the classical marker for T cell clustering, T cells were re-clustered into cytotoxic CD8^+^T cells (CD8-Cytotoxic), exhausted CD8^+^T cells (CD8-exhausted), exhausted CD4^+^T cells (CD4-exhausted), Naïve CD4^+^T cells (CD4-Naïve), Th17 cells (CD4-Th17), Tregs (CD4-Treg), memory CD4^+^T cells (CD4-memory), Natural killer T cells (NK) (Figure 3B).The analysis of constituent ratios for T (Figure 3C), CD4^+^T (Figure 3D) and CD8^+^T (Figure 3E) subsets found that the relative percentages of CD4-memory, CD4-Th17 and CD8-Cytotoxic presented a gradual reduction from PCA-preca-OLK-OSCC, whereas CD4-exhausted, CD8-exhausted and CD4-Treg showed an increase in preca-OLK and OSCC tissues relative to PCA, but no obvious difference between preca-OLK and OSCC tissues in according to Hu'report 13. To further reveal T cells gradual dysfunctionality in oral precancer and cancer, we performed multiple immunofluorescences for Pan-CK, CD3, CD8 and PD-1 in tissue sections of 7 OLK, 8 preca-OLK and 7 OSCC and found that the proportion of CD3^+^CD8^+^PD-1^+^cells (exhausted CD8^+^T cells) in total cells in preca-OLK and OSCC were significantly higher than that in OLK (Figure 3F-G). Furthermore, we used momocle2 to infer the differentiation trajectory of T cells, finding that exhausted T cells were the end state of CD4^+^T and CD8^+^T cells differentiation (Figure 3H-I). These results together imply that T cells display gradual dysfunctionality in oral carcinogenesis, which is consistent with previous studies 4, 13.

### Annotation and distribution of macrophage subpopulations across different lesions

**Figure 4:**
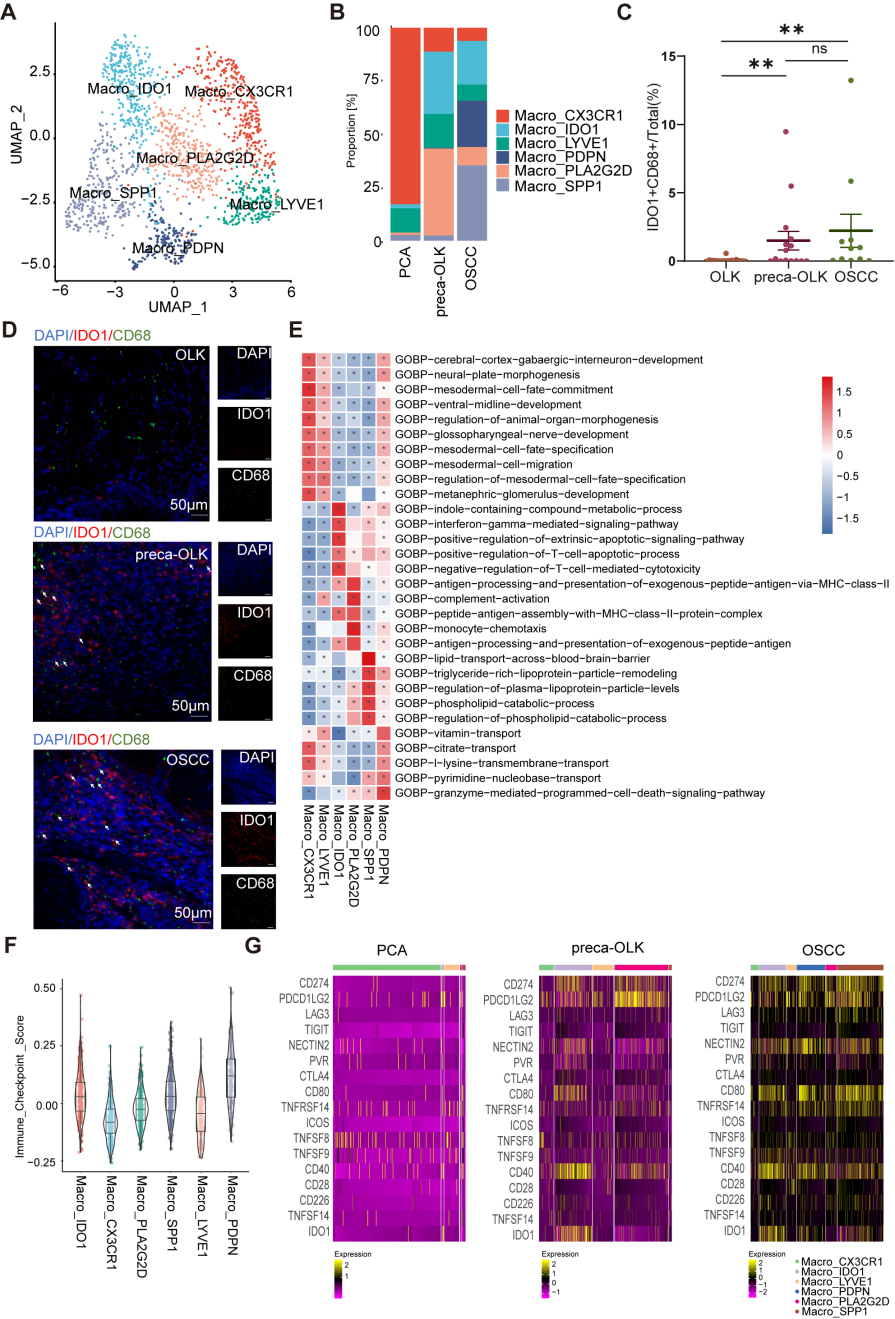
** Annotation and distribution of macrophage subpopulations in PCA, preca-OLK and OSCC (A)** UMAP plot showing the composition of macrophage colored by cluster. **(B)** Bar plot showing proportion of each macrophage subtype in each tissue. **(C)** Statistical analysis comparing the proportions of IDO1^+^CD68^+^cells (IDO1^+^macrophages) in tissues of OLK (n = 25), preca-OLK (n = 15) and OSCC (n = 11). **(D)** Representative IF staining of human tissues (40x). DAPI (blue), IDO1 (red), CD68 (green). Bar, 50 μm. **(E)** Heatmap showing representative GO pathways enrichment of the gene set expressed in macrophage subsets predicted by GSVA. **(F)** Violin plot showing the immunosuppression scoring of macrophage subsets. **(G)** Heatmap showing the expression of immunosuppressive ligand and receptor molecules in macrophage subsets in PCA, preca-OLK and OSCC.

Macrophages exerts obvious affection on carcinogenesis and immunosuppressive status of TME 6-8 and recent scRNA-seq data illustrated that proportions of Macro_NRG1 and Macro_APOE sub-clusters were increased from OLK to OSCC 4. To further explore the heterogeneity of macrophages associated with the transformation of OLK into OSCC, we performed dimensionality reduction clustering to obtain 6 subsets (Figure S3A), which were annotated as Macro-CX3CR1, Macro-IDO1, Macro-LYVE1, Macro-PDPN, Macro-PLA2G2D, Macro-SPP1, respectively, based on their TOP 5 markers (Figure S3B). The distribution of these subsets was shown by UMAP (Figure 4A). The analysis found that the relative percentages of Macro-CX3CR1 and Macro-LYVE1 were higher in PCA compared to preca-OLK and OSCC (Figure 4B), thus, defined by tissue resident macrophages 31. In OSCC, Macro-PDPN and Macro-SPP1 were predominant subsets (Figure 4B). Given that SPP1^+^macrophages are associated with poorer clinical outcomes in cancers 32 and PDPN is related to tumor relapse 33, these two subsets were annotated as TAMs. Macro-IDO1 and Macro-PLA2G2D were dominant subsets in preca-OLK and almost not existent in PCA (Figure 4B), therefore, defined by preca-OLK related cells. More interestingly, the relative percentage of Macro-PLA2G2D obviously reduced while Macro-IDO1 presented decreased tendency but kept some richness in OSCC in relation to preca-OLK.

To further demonstrate the distribution of Macro-IDO1 in carcinogenesis, IDO1 and CD68 immunofluorescence staining was performed in tissue sections of 25 OLK, 15 preca-OLK and 11 OSCC. Comparison of the proportion of IDO1^+^CD68^+^cells (IDO1^+^macrophages) in the total cells (Figure 4C) and in CD68^+^cells (Figure S3C) among the different tissues found that this proportion in preca-OLK was significantly higher than that in OLK. However, there was no significant difference between preca-OLK and OSCC for this proportion in the total cells (Figure 4C-D). All these results suggest that Macro-IDO1 is a main macrophage subcluster in preca-OLK and possessed TAM transformation potentials.

### GSVA GO functional enrichment analysis of macrophage subsets

GSVA GO functional enrichment analysis (Figure 4E) showed that Macro-LYVE1 and Macro-CX3CR1 mainly enriched in human tissue development related functions. Macro-SPP1 was associated with lipid metabolism function, while Macro-PDPN enriched in vitamin, citrate and other transport functions. It has been demonstrated that tumor cells utilize lipid metabolism to support their proliferation, survival, migration, invasion, and metastasis 34 while metabolism of amino acids acts as important signaling molecules indispensable for the growth of cancer cells 35. Thus, the function enrichment of these two subsets possessed the functional characteristics of TAMs. Macro-PLA2G2D functioned in antigen processing presentation, monocyte chemotaxis and complement activation, while PLA2G2D can exert promotional effects on tumor progression and angiogenesis 36. Macro-IDO1 enriched in interferon-γ mediated signaling pathways, immune responses, particularly regulating T cell apoptosis processes, suggesting the immunosuppression function of Macro-IDO1.

To compare immune functions of different macrophage subsets, immune checkpoint scoring of all macrophage subsets was evaluated according to immune checkpoint gene list (Figure 4F, Table S1). Macro-CX3CR1, Macro-LYVE1 and Macro-PLA2G2D scored lower, while Macro-IDO1, Macro-PDPN and Macro-SPP1 scored higher, suggesting that the last three subsets possess immunosuppression efficacy, corresponding with the above analysis. Further analysis of respective immunosuppression related gene (ISRG) expression of macrophage subsets across different lesions (Figure 4G) revealed that the corresponding ISRGs were expressed lowest in PCA, significantly higher in preca-OLK and highest in OSCC, suggesting that macrophages gradually shift to an immunosuppressive phenotype during preca-OLK evolution into OSCC. Interestingly, IDO1 was hardly expressed in Macro-PDPN and Macro-SPP1 in preca-OLK, but highly expressed in partial Macro-PDPN and Macro-SPP1 in OSCC. These results together show that Macro-IDO1 is one of main immunosuppressive macrophage subsets in preca-OLK evolution into OSCC.

### Macro-IDO1 interacts with CD4-exhausted and CD8-exhausted

**Figure 5:**
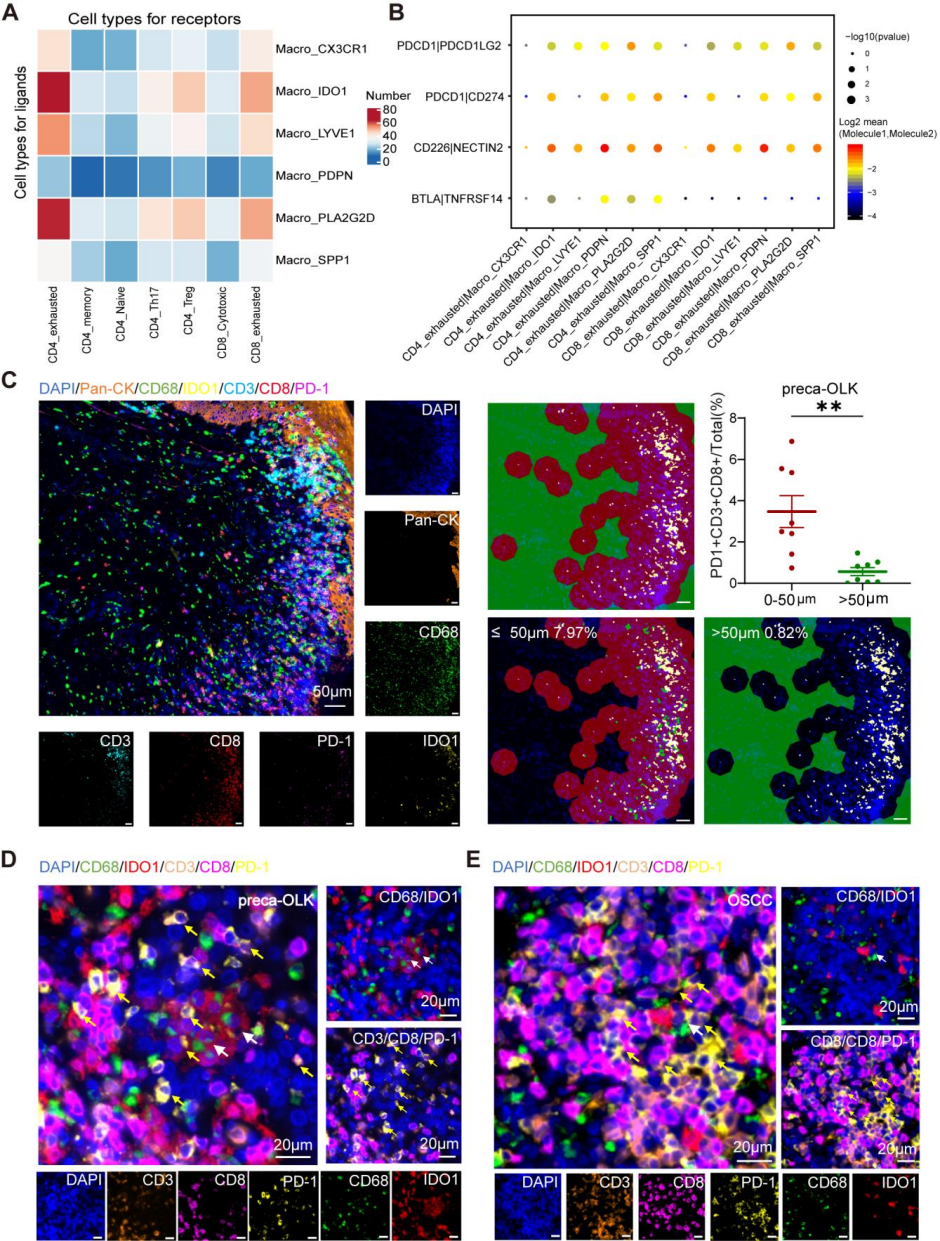
** IDO1^+^ macrophages exert immunosuppressive effects. (A)** Heatmap showing the interacting relationship pairs between macrophage subsets and T cell subsets according to CellPhoneDB analysis in preca-OLK. (B) Dot plot showing the interaction intensity of immune checkpoint ligand/receptors between macrophage subsets and CD4/CD8_exhuausted according to CellPhoneDB analysis. **(C)** mIF results showing the spatial distribution of PD-1^+^CD3^+^CD8^+^ cells (exhausted CD8^+^T cells) around IDO1^+^CD68^+^ cells (IDO1^+^macrophages), red area indicates the area with a radiu≤50 μm, as green area indicates the area with a radiu >50μm from the IDO1^+^CD68^+^ cells (IDO1^+^macrophages). **(D-E)**. Representative image showing the spatial distribution of PD-1^+^CD3^+^CD8^+^ cells (exhausted CD8^+^T cells) in the area ≤50 μm from the IDO1^+^CD68^+^ cells (IDO1^+^macrophages) in preca-OLK **(D)** and OSCC **(E)**. White arrows indicate examples of IDO1^+^CD68^+^cells; yellow arrows indicate examples of PD-1^+^CD3^+^CD8^+^ cells.

To investigate the interaction between macrophages and T cells, we conducted cellphoneDB cell communication analysis on macrophage subsets and T cells in preca-OLK and revealed that Macro-IDO1 and Macro-PLA2G2D had more ligand-receptor counts with CD4-exhausted and CD8-exhausted among all macrophage subsets (Figure 5A). We also performed immune checkpoint-associated ligand/receptor analysis for T cells and macrophage subpopulations and found that among macrophage subpopulations, Macro-IDO1, Macro-SPP1 and Macro-PDPN had the most intimate communication with exhausted T cells by PDCD1/PDCD1LG2, PDCD1/CD274, CD226/NECTIN2, and BTLA/TNFRSF14 axes (Figure 5B, Figure S4A). Considering that in preca-OLK, Macro-SPP1 and Macro-PDPN have a relatively low proportion, while Macro-IDO1 has the highest proportion, the above results suggest that Macro-IDO1 is main macrophage subsets associated with exhausted T cells by immune checkpoint-associated ligand/receptor communication in preca-OLK.

To further uncover the effect of Macro-IDO1 on T cells in carcinogenesis, we performed IDO1, CD68, CD3, CD8, PD-1, and Pan-CK multiple immunofluorescence staining for tissue sections of 7 OLK, 8 preca-OLK and 7 OSCC, and discovered that the proportion of PD-1^+^CD3^+^CD8^+^cells (exhausted CD8^+^T cells) in total cells within 50 μm distant from IDO1^+^CD68^+^ cells (IDO1^+^macrophages) was significantly higher than that outside of 50 μm in both preca-OLK (Figure 5C-D) and OSCC (Figure 5E, Figure S4B). The overall analysis results of the two sets of data are the same (Figure S4C). Furthermore, there was a positive relationship between the proportions of IDO1^+^CD68^+^cells (IDO1^+^macrophages) and the PD-1^+^CD3^+^CD8^+^cells (exhausted CD8^+^T cells) in preca-OLK and OSCC (Figure S4D). This further reveals the relationship between Macro-IDO1 and the CD8-exhausted.

### Macro-IDO1 is derived from tissue resident macrophages and circulating monocytes and activated by IFN-γ-JAK-STAT pathway

**Figure 6:**
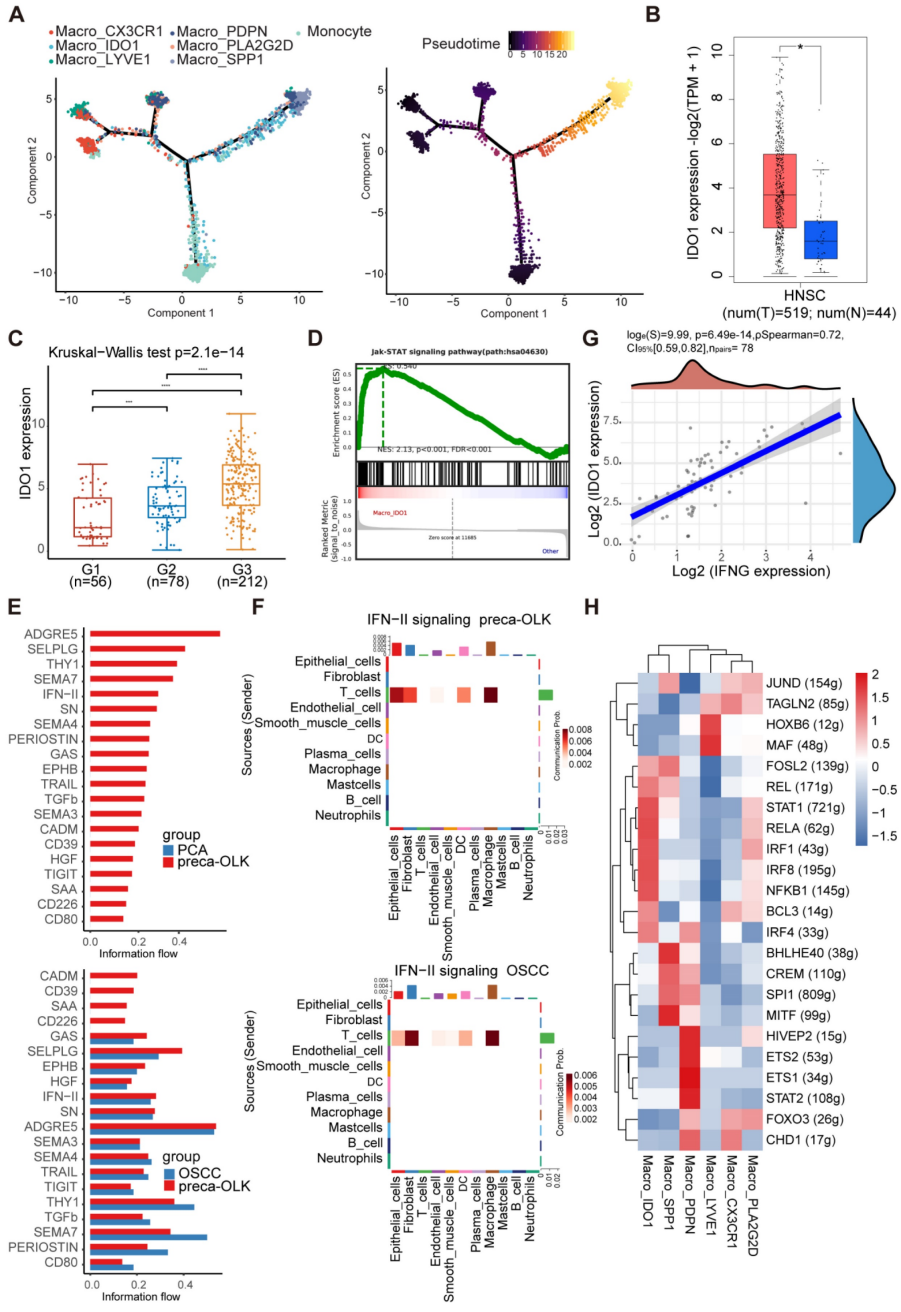
** IDO1 is upregulated in HNSC, and activation of the IFN-γ-JAK-STAT axis is the potential cause for differentiation to IDO1^+^macrophages. (A)** Umap plot showing the differentiation trajectory of macrophages and the distribution of each cell subset on the trajectory. **(B)** Comparison of IDO1 expression between tumor (n = 519) and normal (n = 44) in TCGA database. **(C)** The boxes showing the expression distribution of IDO1 in PCA (G1), OLK (G2) and OSCC (G3) in GEO data. **(D)** Gene set enrichment analysis (GSEA) results showing the enrichment of JAK-STAT gene sets in Macro_IDO1. **(E)** Information flow of feature signaling pathway in different tissues. **(F)** The CellPhoneDB communication analysis demonstrating that IFN-II signaling pathway mediates communication between T cells and macrophages in preca-OLK and OSCC. **(G)** Scatter plot showing the correlation between IFNG and IDO1 expression across 4 independent datasets with OLK in GEO data. **(H)** Heatmap showing RAS activity of regulons in macrophage subtype predicted by SCENIC.

In mice, macrophages can be derived from adult circulating monocytes or from embryonic precursors seeded in tissues during early development 37, 38. To know transformation relationship of macrophage subpopulations, we conducted monocle2 pseudo temporal analysis for monocyte and macrophage subsets (Figure 6A). Results found that monocytes and tissue resident Macro-LYVE1 and Macro-CX3CR were the origins of macrophage evolution, supporting previous study 39, 40. OLK associated Macro-IDO1 and Macro-PLA2G2D were in an intermediate transition state and gradually transited into TAM-like Macro-PDPN and Macro-SPP1. Further analysis of gene expression trends in quasi temporal sequences (Figure S5A) disclosed that Macro-IDO1 characteristic markers IDO1 and LAMP3 maintained a certain abundance in Macro-PDPN and Macro-SPP1 at near end of time series, while Macro-PLA2G2D characteristic markers PLA2G2D and QPRT presented nearly no expression in TAMs. These results further suggest that Macro-IDO1 is derived from tissue resident macrophages and circulating monocytes and possesses TAM transformation potentials.

Then, we wanted to know how Macro-IDO1 was activated. For this inquiry, we first analyzed IDO1 expression in Macro-IDO1 and in carcinogenesis. Single-cell RNA sequencing displayed that CD1E, CXCR2P1, RUFY4, IDO1 and LAMP3 were 5 top genes in Macro-IDO1 (Figure S3B), of which IDO1 can affect T cell immune responses via activation of suppressive Treg cells 41. Data mining analysis of IDO1 expression in cancer and para-cancer tissue using TCGA database showed a higher expression of IDO1 in cancer than in para-cancer tissue (Figure 6B). GEO data (GSE35261, GSE30784, GSE26549, and GSE85195 combination), including 56 normal tissues, 78 OLKs, and 212 OSCCs, revealed a higher expression of IDO1 in OLK and OSCC than in para-cancer tissue (Figure 6C) and a positive relationship between IDO1 and CD68 expression in OLK (Figure S5B). Then, we explored signaling pathway associated with Macro-IDO1 activation. GSEA analysis discovered that the JAK-STAT pathway was enriched in Macro-IDO1 (Figure 6D). It has been reported that IDO1^+^macrophages are modulated by transcription factor STAT1, IFR1 and IFR7 42, 43, while IFN-γ is typical IDO1 upregulation factor 41 and activator of STAT1 pathway 44, 45. Single-cell RNA sequencing found that IFNG expression within total cells gradually increased from PCA-preca-OLK-OSCC (Figure S5C). Information flow by cellchat showed that the IFN-II pathway was opened in preca-OLK and continued to be opened in OSCC. And the pathway related to immunosuppression such as CD80 was opened in preca-OLK and continued to be opened in OSCC (Figure 6E). IFN-II signaling mediated the communication between T cells and macrophages in both preca-OLK and OSCC (Figure 6F). Correlation analysis between IFNG and IDO1 expression in 78 OLK samples obtained from GEO data showed a positive relationship between the two (Figure 6G). Further *in vitro* experiment demonstrated that IFN-γ significantly enhanced IDO1 mRNA expression in THP-1 (Figure S5D). SCENIC analysis of macrophages found that STAT1, IRF1, IRF4 and IRF8 were highly expressed in Macro-IDO1 (Figure 6H). All these results suggest that IFN-γ-JAK-STAT pathway may be regarded as the key factor for IDO1 upregulation in OLK and OSCC.

### IDO1 inhibitor Epacadostat reduces 4NQO induced oral carcinogenesis in mice

**Figure 7:**
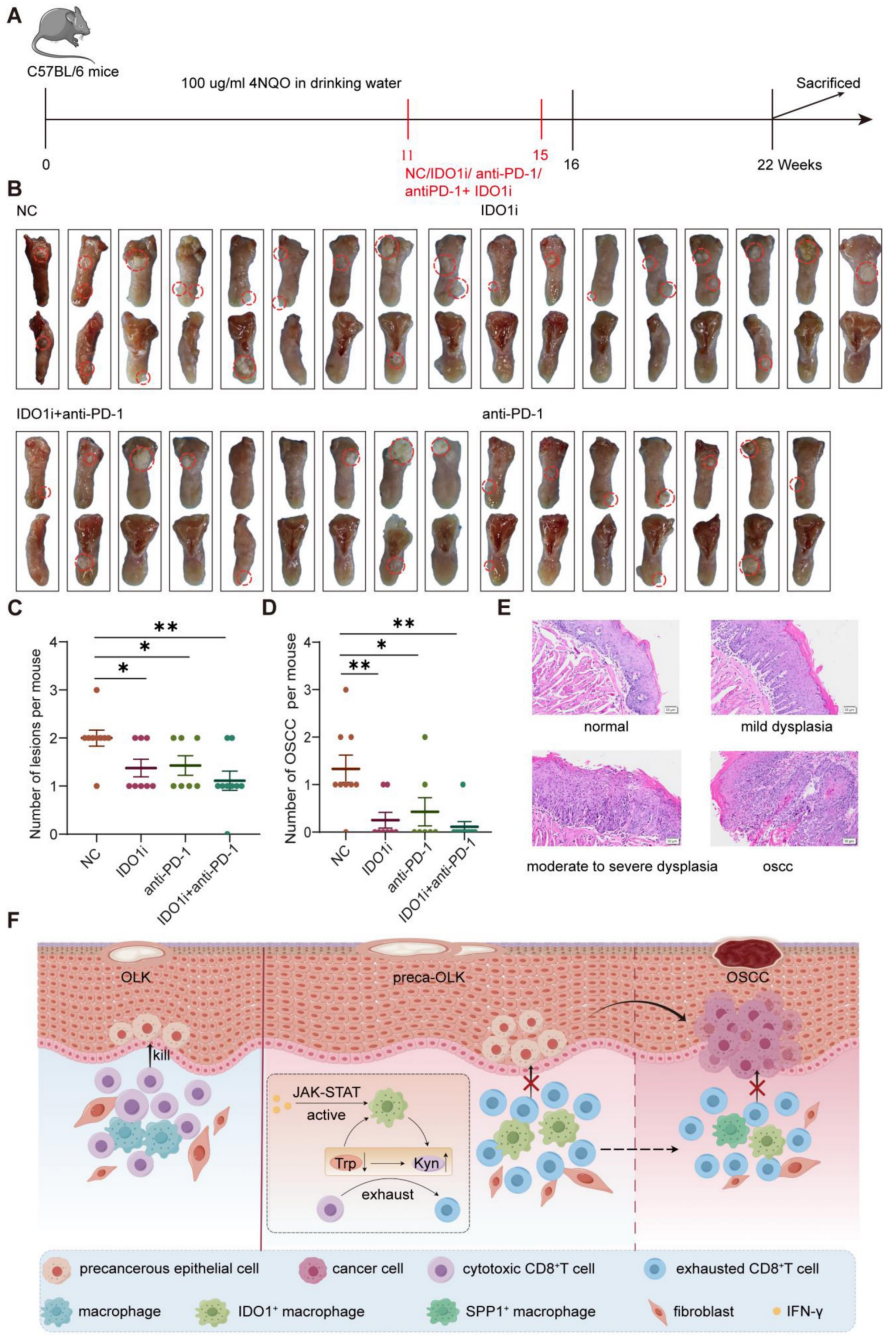
** The IDO1 inhibitor significantly reduces 4NQO induced oral carcinogenesis in mice (A)** Schematic plot showing the induction of OSCC by 4NQO in C57BL/6 mice. (B) Macroscopic lesions on the tongues of each group of mice. The dotted circles indicate macroscopic cauliflower-like lesions. **(C)** Statistical results of macroscopic lesions on the tongues of each group of mice. **(D)** Statistical results of the tongue lesions (carcinoma) in mice of each group. **(E)** Representative microscopic images of typical pathological images following H&E staining. Scale bar: 100 μm. **(F)** Graphical illustration of the working model. Abbreviations: OLK, oral leukoplakia; preca-OLK, precancerous OLK; OSCC, oral squamous cell carcinoma; Trp, tryptophan; Kyn, kynurenine.

A mouse oral squamous cell carcinoma model was established by feeding drinking water containing 100μg/ml 4NQO for 16 consecutive weeks. At 11 weeks, white patches formed on their tongue and no obvious tumor was found. Then, mice were treated for another 4 weeks according to the grouping scheme (untreated control group (NC), IDO1 inhibitor Epacadostat treatment group (IDO1i), anti-PD-1 treatment group (anti-PD-1) and IDO1 inhibitor Epacadostat + anti-PD-1 treatment group (IDO1i+ anti-PD-1)) and at 22 weeks, mice were euthanized to observe the number of tongue lesions (Figure 7A). The visual observation showed that the number of tongue lesions in IDO1i and anti-PD-1 groups was less than that in the NC group (p<0.05), while IDO1i + anti-PD-1 group did not further decrease the number compared to alone IDO1i and anti-PD-1 treatment (Figure 7B-C). HE staining showed that the number of oral OSCCs in three treatment groups was significantly lower than that in the NC group (Figure 7D-E). Of note, although all four groups had exogenous lesions, there was no obvious dysplasia of some of the exogenous structures of three treatment groups (Figure S6). These results prove that IDO1 inhibitor Epacadostat and anti-PD-1 have similar inhibitory effects on the progression of OLK to OSCC.

## Discussion

The present study revealed increased abundance of exhausted T cells and immunosuppressive macrophages and fibroblasts subsets at OLK stage verse the normal counterparts, in line with the recent reports 4, 13, further supporting the notion that immunosuppression plays an important role in transformation of OLK into OSCC. More important, we novelly demonstrated the important role of Macro-IDO1 sub-cluster in shift of precancerous OLK to OSCC, providing a potential target for preventing precancerous legions from transformation into OSCC.

Macrophages play an important role in cancer development and progression, but widely used M1/M2 dichotomy does not embrace macrophage diversity, especially in cancer and carcinogenesis. Although the M2-like phenotype predominates in TAMs and has an established tumor-promoting effect, TAMs always are a mixture of M1-like and M2-like phenotypes, and both protumor 46-48 and anti-tumor role 49, 50 of M1 macrophages has been reported in many studies. TAM differentiation from circulating monocytes appears to be a distinct pathway from the assumed M2 anti-inflammatory (pro-tumoral) status 51. Moreover, TAMs in OLK co-express CD163 and STAT1, the latter is known as an M1-related marker, suggesting that the TAMs involved in OLK exhibit the M1 phenotype in a Th1-dominated microenvironment 52. In OLK with malignant transformation, the number of macrophages expressing CD11c, one of the M1 markers, and expressing CD163, typical M2 marker, was significantly higher than in OLK with nontransformation 53. Thus, macrophages show overlapping phenotypes of M1 and M2 during oral carcinogenesis. Perhaps just because of this, our scRNA-seq data failed to classify macrophages into M1 or M2 subsets based on traditional markers, alternatively, we performed dimensionality reduction clustering to obtain 6 subsets based on their TOP 10 markers. Interestingly, we found that the relative percentage of Macro-IDO1 presented decreased tendency and Macro-PLA2G2D obviously reduced in OSCC in relation to OLK, therefore, these two subsets were defined by OLK malignant transformation related cells.

PLA2G2D, a kind of lipid metabolism-associated protein, can be secreted and involved in inflammatory responses through hydrolyzing membrane phospholipids and releasing unsaturated fatty acids 54. PLA2G2D has been shown to be significantly correlated with the infiltration of immune cells, especially CD8^+^T cells and macrophages and with increased cytotoxicity and favorable response to immune checkpoint blockade (ICB) therapy in cervical squamous cell carcinoma (CSCC) 55. A similar report pointed out that PLA2G2D positively correlated with CD8^+^T cells, M1 macrophages and CD4^+^ memory activated T cells and negatively correlated with M2 macrophages, suggesting its potential anti-tumor effect 56. Nevertheless, PLA2G2D is also reported to plays a key role in non-small cell lung cancer angiogenesis through aerobic glycolysis 36. Our study found that the proportion of Macro-PLA2G2D sub-cluster elevated in OLK but decreased in OSCC, implying that the role of PLA2G2D in precancer lesions remains to be elucidated.

Studies reveal that IDO1 is an immunosuppressant in the TME and related to tumor progression 57. IDO1 regulates tryptophan metabolism by catabolizing tryptophan to kynurenine, and reduced tryptophan levels are associated with poor outcomes among multiple cancer types 58-60. Tryptophan catabolism is suggested as playing an important role in resistance to immunotherapy 61, 62. In preclinical models, heightened activity of the Trp-Kyn-AhR pathway has been linked to impairment of antitumor immunity and tumor growth 57, 63. Accumulation of kynurenines induce effector T cell arrest and lead to binding of AhR. This results in nuclear translocation and promotion of Foxp3 transcripts and IL-10, eventually producing regulatory T cell populations 64-67. Taken together, ample evidence supports the role of IDO1 as an immunosuppressive molecule in the tumor microenvironment. However, the production and role of IDO1 in oral carcinogenesis is uncertain. Our study not only showed IDO1^+^macrophages being main macrophage subset and the elevated IDO1 expression in OLK and OSCC, but also demonstrated its potential immunosuppressive function, indicating that Macro-IDO1 plays a key role in malignancy transformation of oral precancer lesions through immunosuppression.

It is known that in mice, macrophages can be derived from adult circulating monocytes or from embryonic precursors seeded in tissues during early development 37, 38. Translation of these findings to the human is challenging, but scRNA-seq permits overcoming such challenges 68. Our findings by using momocle2 to infer the differentiation trajectory of Macro-IDO1 sub-cluster showed that, consistent with the above, IDO1^+^ macrophages were mainly originated from tissue resident macrophages and circulating monocytes and some cells were in the middle status, and gradually transformed into TAMs.

It has been reported that IDO1^+^macrophages are modulated by transcription factor STAT1, IFR1 and IFR7 42, 43, while IFN-γ is typical IDO1 upregulation factor 41 and activator of STAT1 pathway 62, 63. In our study, Macro-IDO1 mainly enriched in JAK-STAT pathway and highly expressed STAT1, IFR1, IRF4 and IRF8. IFN-II pathway was opened in preca-OLK and continued to be opened in OSCC and mediated the communication between T cells and macrophages in both preca-OLK and OSCC. Further *in vitro* experiment demonstrated that IFN-γ significantly enhanced IDO1 mRNA expression in THP-1. All these results suggest that IFN-γ-JAK-STAT pathway may be regarded as the key factor for IDO1 upregulation in OLK and OSCC.

Not all oral leukoplakia gets transformed into oral cancer and moreover, no reliable markers can be used to predict the potential malignancy shift. Thus, the use of the leukoplakia tissues with more clear malignant transformation potential may better unveil the molecular events in oral carcinogenesis. For example, Ries et al compared PD-1 and PD-L1 expression in OLK that had undergone malignant transformation within 5 years and in OLK without malignant transformation, finding that increased levels of PD-1 and PD-L1 are related to malignant transformation in OLP and may represent a promising prognostic indicator to determine the risk of malignant progression of OLK within 5 years 69. Sun et al collected tissue samples simultaneously containing early OSCC, OLK, and matched normal region and discovered multiple cellular landscapes and roles of precancerous lesions underlying OSCC initiation by single-cell and spatial dissection technique 4. In our immunofluorescence study, we utilized tissue sections from 25 non-malignant OLK (OLK) and 15 malignant OLK (preca-OLK) and 11 OSCC to demonstrate that in preca-OLK, there was the higher proportion of IDO1^+^ macrophages and exhausted CD8^+^T cells than in OLK. In our single-cell RNA sequencing, tissue samples simultaneously contained OSCC, OLK, and matched normal region. Therefore, tissue samples used in the present and other studies 4, 6 may more truly reflect the role of OLK in carcinogenesis compared to overall leukoplakia.

In summary, through single-cell RNA sequencing and further validation using multicolor immunofluorescence staining, TCGA/GEO database analysis and the *in vitro*/*in vivo* experiments, this study shows that immunosuppressive TME has formed at OLK stage before transformation into OSCC, as evidenced by increased immunosuppressive cells such as exhausted T cells and some subsets of macrophages and fibroblasts, further supporting the notion that immunosuppression plays an important role in transformation of OLK into OSCC. More importantly, we novelly demonstrated the important role of Macro-IDO1 sub-cluster in shift of precancerous OLK to OSCC, providing a potential target for preventing precancerous legions from transformation into OSCC. However, our study has several limitations, including, but not limited to: 1) The number of clinical samples for single-cell RNA sequencing was limited; 2) The lack of prognostic data for OLK in the database, as well as the long follow-up time required to obtain prognostic data for leukoplakia in the clinic, results in a lack of clinical evidence for the prognostic impact of IDO1^+^ macrophages on oral leukoplakia, which needs to be validated in further prospective clinical studies.

## Supplementary Material

Supplementary figures and table.

## Abbreviations

OSCC: oral squamous cell carcinoma
OLK: oral leukoplakia
scRNA-seq: single-cell RNA sequencing
preca-OLK: precancerous OLK
PCA: paracancerous tissue
CAF: cancer-associated fibroblast
PD-1: programmed cell death receptor 1
PD-L1: programmed cell death ligand 1
UMAP: uniform manifold approximation and projection
TME: tumor microenvironment
TAM: tumor-associated macrophage
IDO1: indoleamine 2,3 dioxygenase 1
Treg: regulatory T cell
GSVA: gene set variation analysis
GSEA: gene set enrichment analysis
GO: gene ontology
ISRG: immunosuppression related gene
TCGA: the cancer genome atlas
GEO: gene expression omnibus
