# Supplementary material for: Single-cell RNA sequencing highlights the immunosuppression of IDO1+ macrophages in the malignant transformation of oral leukoplakia
Source: Theranostics. 2024 Aug 12;14(12):4787–805. doi: 10.7150/thno.99112 (PMC11373622; doi:10.7150/thno.99112)
Supplement: Supplementary file 1 — Supplementary figures and table. [file thnov14p4787s1.pdf]

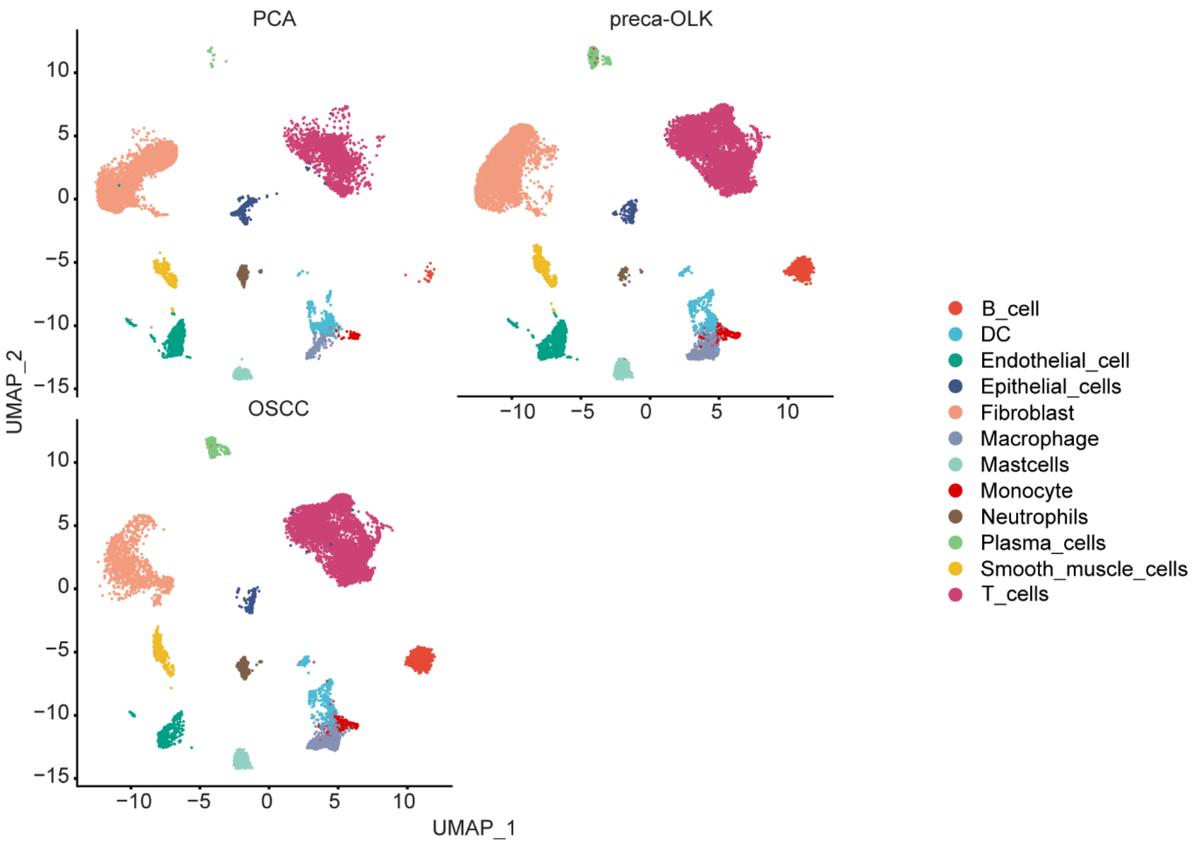

Figure S1. (related to Figure 1) UMAP plots of cell clusters respectively from PCA, preca-OLK and OSCC.

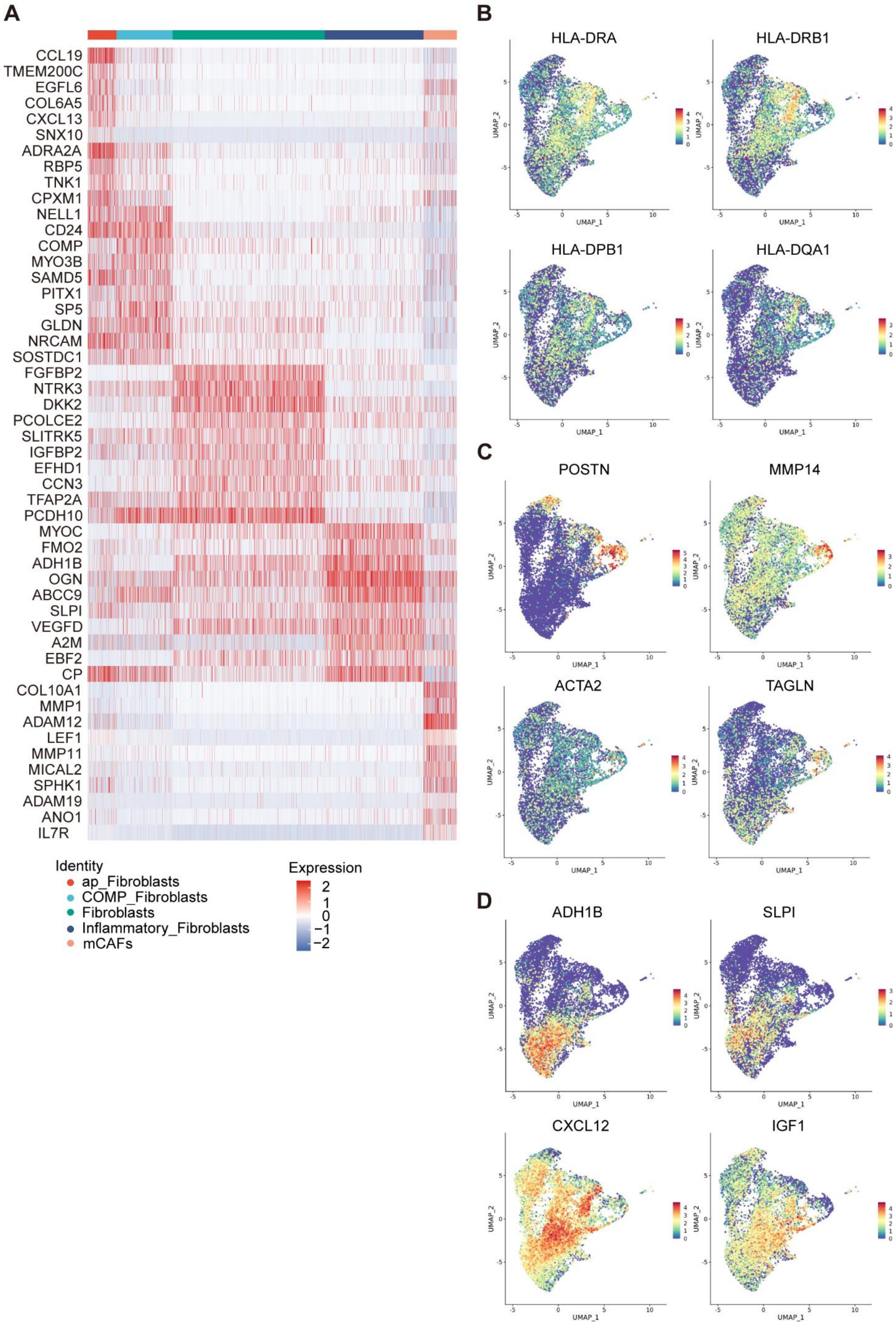
A
CCL19
TMEM200C
EGFL6
COL6A5
CXCL13
SNX10
ADRA2A
RBP5
TNK1
CPXM1
NELL1
CD24
COMP
MYO3B
SAMD5
PITX1
SP5
GLDN
NRCAM
SOSTDC1
FGFBP2
NTRK3
DKK2
PCOLCE2
SLITRK5
IGFBP2
EFHD1
CCN3
TFAP2A
PCDH10
MYOC
FMO2
ADH1B
OGN
ABCC9
SLPI
VEGFD
A2M
EBF2
CP
COL10A1
MMP1
ADAM12
LEF1
MMP11
MICAL2
SPHK1
ADAM19
ANO1
IL7R
Identity
ap_Fibroblasts
COMP_Fibroblasts
Fibroblasts
Inflammatory_Fibroblasts
mCAFs
Expression
2
1
0
−1
−2
B
HLA-DRA
HLA-DRB1
HLA-DPB1
HLA-DQA1
C
POSTN
MMP14
ACTA2
TAGLN
D
ADH1B
SLPI
CXCL12
IGF1
UMAP_1
UMAP_2

Figure S2. Expression levels of selected known marker genes of fibroblast subsets. (related to Figure 2) (A)Heatmap showing Top10 marker genes in macrophage subsets. Expression levels of selected known marker genes of ap-Fibroblasts (B) mCAFs (C) Inflammatory Fibroblasts (D) across fibroblasts illustrated in UMAP plots.

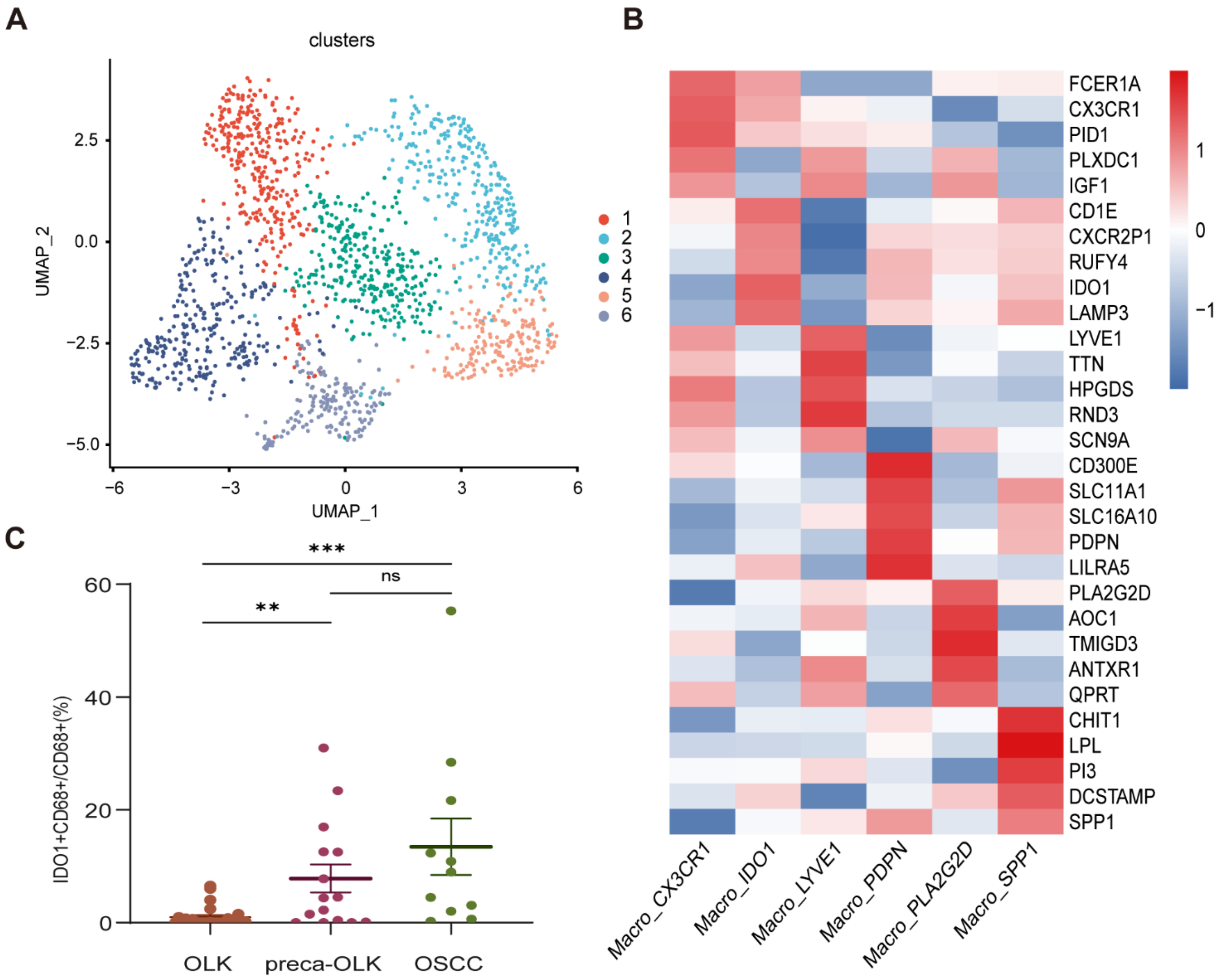

Figure S3. Distribution and marker genes of macrophage subpopulations in PCA, preca-OLK and OSCC. (related to Figure 4) (A) UMAP plot of individual macrophage cells. (B) Heatmap showing highly expressed marker genes in macrophage subsets. (C) Statistical analysis comparing the proportions of IDO1^{+}CD68^{+} cells (IDO1^{+}macrophage) among CD68^{+} cells (macrophages) in tissue of OLK (n = 25), preca-OLK (n = 15) and OSCC (n = 11).

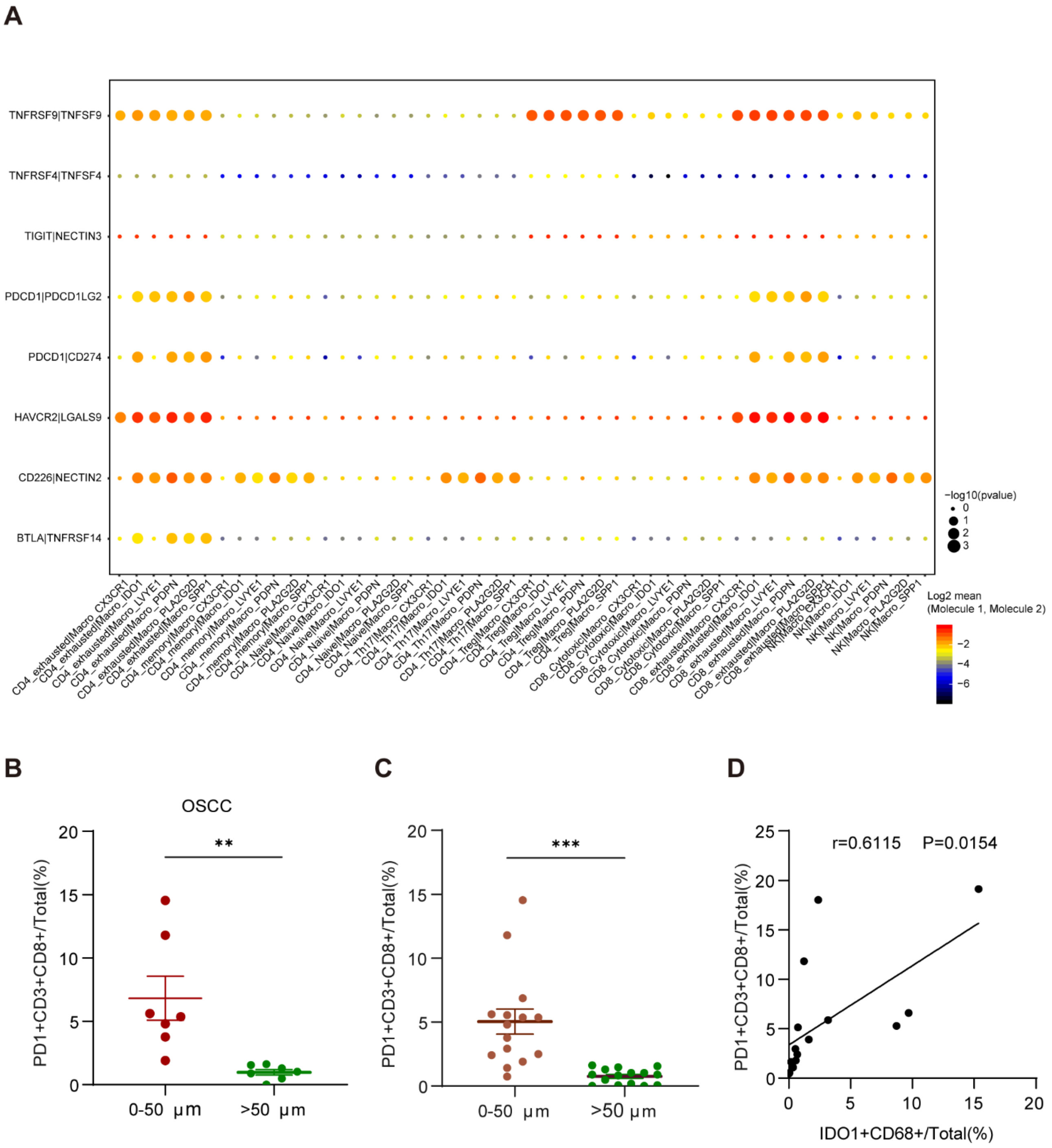

Figure S4. IDO1⁺ macrophages exert immunosuppressive effects (related to Figure 5) (A) Dot plot showing the interaction intensity of immune checkpoint ligand/receptors between macrophage subsets and T cell clusters according to CellPhoneDB analysis. (B-C) Statistical analysis comparing the proportions of in the area ≤ 50μm and > 50μm from the $IDO1^+CD68^+$ cells ($IDO1^+$ macrophages) in OSCC (B) or all tissues (C). (D) Scatter plot showing the correlation between $PD1^+CD3^+CD8^+$ cells (exhausted $CD8^+$T cells) and $IDO1^+CD68^+$ cells ($IDO1^+$ macrophages) in preca-OLK and OSCC tissues.

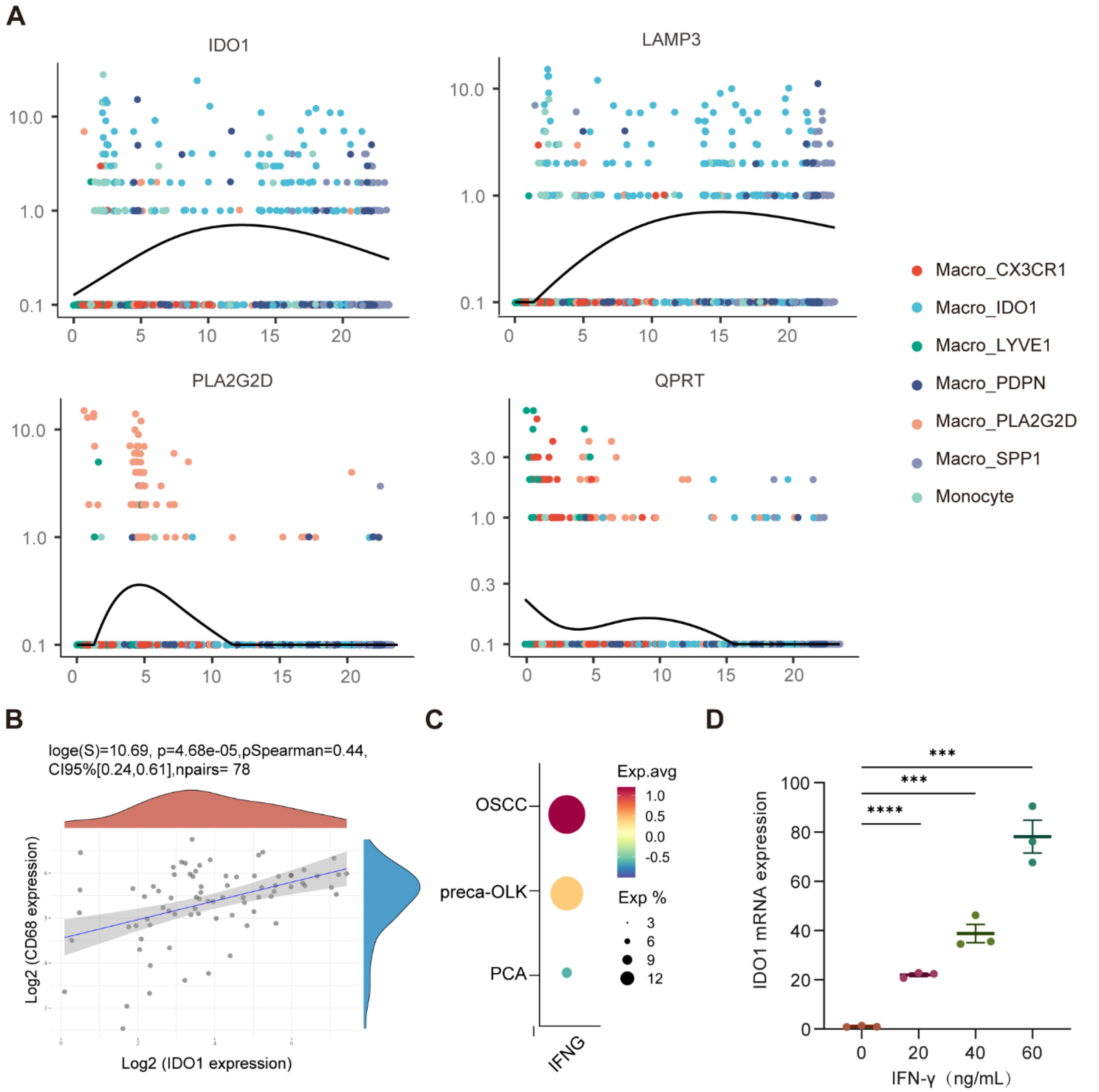

Figure S5. The upregulation of IDO1 is typically mediated by IFN-γ. (related to Figure 6) (A) Changes in gene expression trends over time in different macrophage subsets. (B) Scatter plot showing the correlation between IDO1 and CD68 expression across 4 independent datasets with OLK. (C) Dot plot showing expression of IFNG in different tissues. (D) Stimulation with IFN-γ increased the expression of IDO1 in THP1 monocytic cells. (**$p < 0.01$ ***$p < 0.001$, ****$P < 0.0001$).

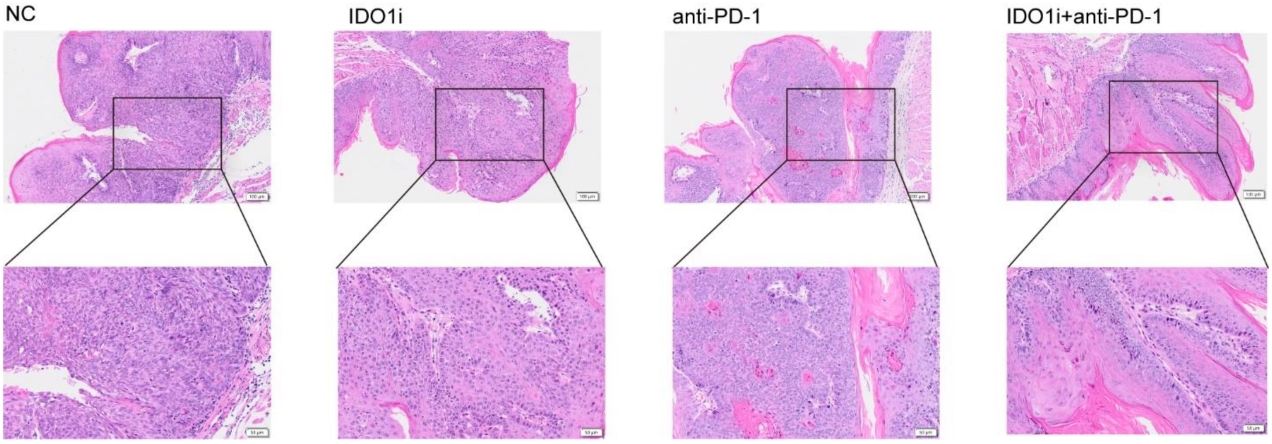

Figure S6. Representative HE images of macroscopic cauliflower-like lesions of mouse tongue in each group. (**related to Figure 7**) Representative HE images of macroscopic cauliflower-like lesions of mouse tongue in each group. Scale bars: 400 µm (top) and 50 µm (bottom).

Supplementary Table S1

gene ID
PDCD1
CD274
PDCD1LG2
HAVCR2
LGALS9
CEACAM1
LAG3
FGL1
TIGIT
NECTIN2
PVR
CTLA4
CD80
CD86
LAIR1
TNFRSF14
ICOS
TNFSF4
TNFSF8
TNFSF9
CD40
CD27
CD28
CD226
TNFSF14
IDO1
